# IL-4-Responsive B Cells Are Detrimental During Chronic Tuberculosis Infection in Mice

**DOI:** 10.3389/fimmu.2021.611673

**Published:** 2021-06-15

**Authors:** Suraj P. Parihar, Mumin Ozturk, Maxine A. Höft, Julius E. Chia, Reto Guler, Roanne Keeton, Ilana C. van Rensburg, Andre G. Loxton, Frank Brombacher

**Affiliations:** ^1^ International Centre for Genetic Engineering and Biotechnology (ICGEB), Cape Town Component, Cape Town, South Africa; ^2^ Division of Immunology and South African Medical Research Council (SAMRC) Immunology of Infectious Diseases, Institute of Infectious Diseases and Molecular Medicine (IDM), Department of Pathology, Faculty of Health Sciences, University of Cape Town, Cape Town, South Africa; ^3^ Division of Medical Microbiology, Department of Pathology, Faculty of Health Sciences, Wellcome Centre for Infectious Diseases Research in Africa (CIDRI-Africa) and Institute of Infectious Diseases and Molecular Medicine (IDM), University of Cape Town, Cape Town, South Africa; ^4^ AFGrica Medical Mycology Research Unit, Department of Pathology, Faculty of Health Sciences, Institute of Infectious Diseases and Molecular Medicine (IDM), University of Cape Town, Cape Town, South Africa; ^5^ Department of Pathology, Faculty of Health Sciences, Wellcome Centre for Infectious Diseases Research in Africa (CIDRI-Africa) and Institute of Infectious Diseases and Molecular Medicine (IDM), University of Cape Town, Cape Town, South Africa; ^6^ Division of Medical Virology, Department of Pathology, Faculty of Health Sciences, Institute of Infectious Diseases and Molecular Medicine (IDM), University of Cape Town, Cape Town, South Africa; ^7^ DST-NRF Centre of Excellence for Biomedical Tuberculosis Research, South African Medical Research Council Centre for Tuberculosis Research, Division of Molecular Biology and Human Genetics, Faculty of Medicine and Health Sciences, Stellenbosch University, Cape Town, South Africa

**Keywords:** B cells, TB, Mice (balb/c), human, IL-4RA

## Abstract

In tuberculosis, T cell-mediated immunity is extensively studied whilst B cells received limited attention in human and mice. Of interest, *Mycobacterium tuberculosis* (*Mtb*) does increase IL-4 Receptor-alpha (IL4Rα) expression in murine B cells. To better understand the role of IL4Rα signalling in B cells, we compared wild type mice with B cell-specific IL4Rα deficient mice (mb1^cre^IL-4Rα^-/lox^ mice). Chronic *Mtb* aerosol infection in mb1^cre^IL-4Rα^-/lox^ mice reduced lung and spleen bacterial burdens, compared to littermate (IL-4Rα^-/lox^) control animals. Consequently, lung pathology, inflammation and inducible nitric oxide synthase (iNOS) expression were reduced in the lungs of mb1^cre^IL-4Rα^-/lox^ mice, which was also accompanied by increased lung IgA and decreased IgG1 levels. Furthermore, intratracheal adoptive transfer of wild-type B cells into B cell-specific IL4Rα deficient mice reversed the protective phenotype. Moreover, constitutively mCherry expressing *Mtb* showed decreased association with B cells from mb1^cre^IL-4Rα^-/lox^ mice *ex vivo*. In addition, supernatants from *Mtb*-exposed B cells of mb1^cre^IL-4Rα^-/lox^ mice also increased the ability of macrophages to produce nitric oxide, IL-1β, IL-6 and TNF. Together, this demonstrates that IL-4-responsive B cells are detrimental during the chronic phase of tuberculosis in mice with perturbed antibody profiles, inflammatory cytokines and *tnf* and *stat1* levels in the lungs.

## Introduction

B cells are well established as antibody-producing cells critical for the humoral arm of adaptive immunity against a variety of infections. Emerging results uncover more complex antibody-independent involvement of B cells in regulation and effector functions, influencing the outcome of the disease. B cells can process and present antigens, act as accessory cells and produce cytokines that prime other immune cells critical for immunity against infections (1, 2). Like T cells, studies showed that B cells are classified as “regulatory” and “effector” B cell subsets based on the cytokines they produce (3). Regulatory B cells produce TGF-β and IL-10, important in colitis (4), arthritis (5) and allergic airway inflammation (6, 7) and tuberculosis (8). Effector Be1 cells produce IFN-γ (9), IL-12, TNF during Th1 driving infections such as *Leishmania major* (10), *Toxoplasma gondii* (3). Effector Be2 (11) cells produce IL-2, IL-4, IL-13 during Th2 driving *Heligmosomoides polygyrus* (12) and *Nippostrongylous brasiliensis* (13) infections. Thus, B cell-driven cytokines drive host beneficial or detrimental response during type 1 and type 2 infections.

In tuberculosis, B cells are present in lymphoid clusters in mouse (14), non-human primate (15) and human tuberculous granulomas (14, 16, 17). B cells participate in orchestrating granuloma formation is revealed by studies of targeted depletion using either anti-CD20 antibody/rituximab (18) or B cell-deficient mice (19, 20). However; the variations during tuberculosis outcome ranges from B cells being redundant (21), delay immune responses (22) and control lung pathology (19). Moreover, studies showed the role of B cells in granulomatous inflammatory responses by controlling neutrophilia and Th17 responses (23), IL-10 regulation and consequent host protection (19, 24). However, in cynomolgus macaques, B cell depletion using rituximab showed no influence on the outcome of tuberculosis disease (25). Such global depletion approaches masked distinct B cell functions, the local effects of B cells, the contribution of B cell subsets and their secreted cytokines in shaping immune responses necessary for the control of tuberculosis. Hence, such broad approaches from these studies failed to identify a major role of B cells in tuberculosis. Apart from B cells, in patients with active pulmonary tuberculosis, IL-4 secretion from BAL cells revealed a strong association with acid-fast *Mycobacterium tuberculosis* bacilli staining in sputum smear (26), suggesting a permissive Th2 environment at the site of infection. In some studies, IL-4 was shown to predict the development of active TB disease in exposed healthcare workers and household contacts (27, 28). We have previously shown that the disruption of IL-4Rα signalling in macrophages/neutrophils did not play a role in TB disease progression in mice (29). The ability of *Mtb* to induce Arginase 1 independent of IL-4Rα signalling contributed to the lack of phenotype in these mice. In a recent study, recombinant IL-4 impaired containment of *Mtb* in monocyte-derived macrophages associated with the expansion T_reg_ population amongst T_eff_ cells (30). The effect of IL-4 signalling on lymphocytic cells may be more profound than myeloid cells in tuberculosis.

Therefore, we hypothesized that the ablation of IL-4Rα signalling on B cells specifically influences the immune response and the outcome of tuberculosis disease. The present study used BALB/c mice lacking IL-4Rα specifically on B cells, mb1^cre^IL-4Rα^-/lox^, while maintaining intact receptor signalling on other cells (31). We show that the B cells lacking IL-4Rα have decreased mycobacterial burdens and lung pathology during the chronic tuberculosis infection. Importantly, adoptive transfer of IL-4Rα-sufficient B cells from wild-type donor mice abolished the protective effect in mb1^cre^IL-4Rα^-/lox^ mice. We uncovered IL-4Rα deletion on B cells decreased *tnf* and *stat1* expression and also dampened lung IFN-β production. Mechanistically, we show that the absence of IL-4Rα on B cells increased macrophage inflammatory response *ex vivo*.

## Methods

### Mice

Wild-type (BALB/c), littermate control (IL-4Rα^-/lox^) and B cell-specific IL-4Rα deficient mice (mb-1^cre^IL-4Rα^-/lox^) on a BALB/c background (8-12 weeks) were kept under specific-pathogen-free conditions in individually ventilated cages. The genotypes of the mice were confirmed by PCR analysis of the DNA from tail biopsies. All experiments were performed in accordance with the South African National Guidelines and University of Cape Town of practice for laboratory animal procedures.

### *Mtb* Culture and Aerosol or Intranasal Infection in Mice

*Mycobacterium tuberculosis* H37Rv was grown in Middlebrook 7H9 broth as described previously (29). Prior to infection, stock solutions of *Mtb* were thawed, washed once with phosphate-buffered saline and inoculum was prepared in sterile saline. Aerosol infection was performed using an inhalation exposure system (model A4224, Glas-Col). To infect mice with a low dose of 100 CFU/lung, animals were exposed for 40 min to an aerosol generated by nebulizing approximately 6 ml of a suspension containing 2.4x10^7^ live bacteria. Similarly, for intranasal infection, 25µl per nostril was administered in anaesthetized mice to achieve the indicated dose. After infection, the inoculum was also plated to determine the change in the inoculum. Infection dose was checked at one day post-infection by determining the bacterial load in the lungs of four infected mice.

### Determination of Mycobacterial Load, Histopathology and Immunohistochemistry

Mycobacterial loads in lungs and spleen of *Mtb*-infected mice were determined at different time points post-infection as previously described (29). Lungs of *Mtb*-infected mice were fixed with 4% phosphate-buffered formalin, and 3 μm-thick sections were stained with either H&E or rabbit anti-mouse antibody specific for iNOS (Abcam) or rabbit anti-mouse IgA antibody (Abcam). Detection was performed using HRP-labelled anti-rabbit antibody (Dako) followed by 3, 3’-diaminobenzidine substrate (Dako). The lung images and lesion areas, iNOS and IgA positive areas were acquired in Nikon 90i Eclipse widefield microscope and quantified using NIS elements.

### Lung Immune Cell Populations

Single-cell suspensions of the lungs were prepared as previously described (29). 1x10^6^ cells were then subjected to staining for B cells (CD3^-^CD19^+^), CD4 T cells (CD19^-^CD3^+^CD4^+^), CD8 T cells (CD19^-^CD3^+^CD8^+^), macrophages (CD11c^-^CD11b^+^MHCII^+^), dendritic cells (CD11b^-^CD11c^+^MHCII^+^) and neutrophils (SiglecF^-^CD11c^-^Gr-1^+^) in presence of 1% rat serum and 10μg/ml FcyR blocking antibody for 30min on ice. Similarly, lung B cell subsets were analyzed as B-1a (CD19^+^B220^+^CD43^+^CD5^high^IgM^+^), B-1b (CD19^+^B220^+^CD43^+^CD5^low^IgM^+^), B-2 (CD19^+^B220^+^CD43^-^IgM^+^IgD^+^) B-10 (CD19^+^B220^+^CD43^+^CD5^+^CD1d^+^), Plasma (CD19^+^CD138^+^MHCII^low^CD44^high^), Plasmablast (CD19^+^CD138^+^MHCII^+^CD44^high^), IgM (CD19^+^B220^+^CD43^-^IgM^+^) and IgD (CD19^+^B220^+^IgD^+^) B cells. Cells were washed then fixed in 2% paraformaldehyde overnight and acquired by FACS LSRII (BD Pharmingen) and analysed by FlowJo (TreeStar, US). Gating strategies are provided in **Supplementary Figures 2** and **3**. Flow cytometry antibody details are provided in **Supplementary Table 1**.

### Analysis of Cytokines and Antibodies in the Lung Homogenates

Lung homogenates were analysed for the IFN-β (BioLegend), IL-6 (BD Biosciences), IL-12p40 (BD BioSciences) and IL-10 (BD BioSciences) by ELISA according to manufacturers’ instructions. Total IgA, IgE and IgG1 (Southern Biotech) levels are measured in lung homogenates by coating with unlabelled goat anti-mouse antibodies (1:500 dilution) and detection with alkaline phosphatase-conjugated rat anti-mouse antibodies (1:1000 dilution).

### Adoptive Transfer of B Cells

A single-cell suspension of spleen from wild-type mice was prepared to stain total spleen cells using CD3, CD19 and B220 (BD Biosciences) surface markers. Double-positive B cells (CD3^-^CD19^+^B220^+^) were sorted (purity ~98%) using BD FACSAria. 1 million B cells were then transferred intranasally in mb-1^cre^IL-4Rα^-/lox^ mice. Two days after the transfer, mice were infected with *Mtb* and sacrificed at 18 weeks after infection.

### Gene Expression in Sorted B Cells From Chronic *Mtb* Infection

Single-cell suspensions of the lungs were prepared as described previously (29). Cells were stained for B cells (CD3^-^CD19^+^) and sorted with BD FACSJazz instrument. Cells were lysed in 0.5 ml of Qiazol (Qiagen) and total RNA was extracted by RNAeasy Micro kit (Qiagen). Total RNA was transcribed into cDNA using Transcriptor First Strand cDNA Synthesis Kit (Roche) according to the manufacturer’s instructions. Real-time qPCR was performed with LightCycler^®^ 480 SYBR Green I Master mix in LightCycler^®^ 480 II (Roche). Quantitative expression analysis of *Ifnb, il10, il6, Tnf* and *Stat1* were normalized against the housekeeping gene *Hprt*, primer sequences are shown in **Supplementary Table 2**.

### B Cell Infection by mCherry *Mtb* and Supernatant Transfer to *Mtb*-Infected Macrophages

CD19^+^ bead (Miltenyi) sorted cells from naïve spleens of control littermate (IL-4Rα^-/lox^) and B cell-specific IL-4Rα deficient mice (mb1^cre^IL-4Rα^-/lox^) were exposed to constitutively mCherry expressing *Mtb* for 24 hours at a multiplicity of infection 2. B cells were later analysed for mCherry, MHCII and CD124 expression by BD Fortessa. B cell supernatants were then filtered with 0.2 µm filters to remove any extracellular *Mtb*. The supernatants were transferred to *Mtb*-infected bone marrow-derived macrophages (MOI:0.5) and incubated for 3 days. Macrophage supernatants were then analysed for the indicated cytokines by ELISA and nitric oxide by Griess reagent assay.

### IL4RA and Arginase Expression on Peripheral B Cells Isolated From TB Cohort

We enrolled newly diagnosed, untreated TB cases from the clinics in Ravensmead and Uitsig, Cape Town. The participants were treated with standard anti-TB drugs for six months by the clinic. For this study, we took blood at diagnosis and at the end of anti-TB treatment after 23 weeks. We also included healthy participants from the same community. Both the TB cases and the healthy controls were HIV negative. B cells were isolated by CD19 MACS beads from peripheral blood mononuclear cells and RNA was extracted using the RNEasy^®^ Mini Kit (Qiagen, Germany) according to manufacturer’s instructions. RNA was stored at -80°C prior to perform the cDNA synthesis (First Strand Kit (Qiagen, Germany) for quantitative PCR analysis.

### Study Approval

The protocol was approved by the Animal Ethics Committee (AEC Permit Number: 015/040), Faculty of Health Sciences, University of Cape Town, Cape Town, South Africa. Participant recruitment and follow up was approved by the Human Research Ethics Committee of Stelllenbosch University (N10/01/013). Written informed consent was obtained from all study participants.

### Statistics

Data are represented as mean values ± SEM. Statistical analysis was performed using Student’s *t*-test, two-tailed, Welch’s correction with unequal variance and ordinary one-way ANOVA, defining differences between mb1^cre^IL-4Rα^-/lox^ and IL-4Rα^-/lox^ as significant *, *P* ≤ 0.05; **, *P* ≤ 0.01; ***, *P* ≤ 0.001.

## Results

### IL-4Rα Deletion on B Cells Decreased *Mtb* Burdens and Lung Pathology During Chronic Infection in Mice

**Figure 1:**
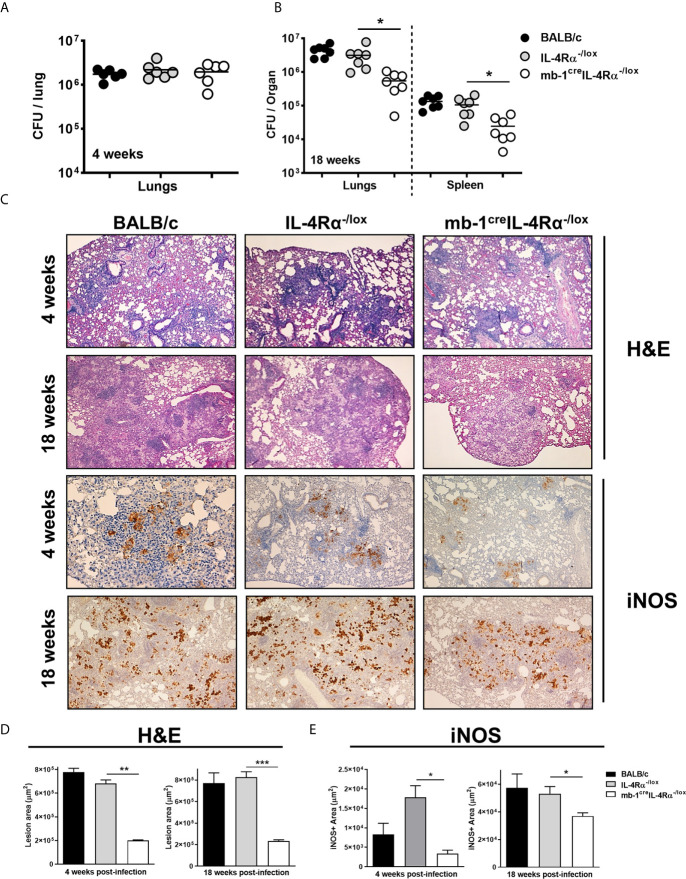
Deletion of IL-4Rα on B cells decreased mycobacterial burdens and lung pathology during *Mtb* infection. Wild-type (BALB/c), littermate controls (IL-4Rα^-/lox^) and B cell-specific IL-4Rα deficient mice (mb-1^cre^IL-4Rα^-/lox^) were infected *via* aerosol inhalation with a dose of 200 CFU H37Rv. **(A)** Mycobacterial burdens in the lungs at 4 weeks post-infection. **(B)** Lung mycobacterial burden and dissemination in the spleen at 18 weeks post-infection. **(C)** Representative histology images of lung sections stained with H&E and iNOS at 4 and 18 weeks post-infection (Original magnification: 10X). **(D, E)** Quantification of lesion area and iNOS positive area in the lungs at 4 and 18 weeks post-infection. Data are shown as mean ± SEM of n = 6 mice/group and representative of two independent experiments, analysed by unpaired, student’s t-test versus littermate control, *p < 0.05, **p < 0.01 and ***p < 0.001.

We assessed the role of IL-4Rα signalling on B cells using wild-type (BALB/c), littermate control (IL-4Rα^-/lox^) and B cell-specific IL-4Rα (mb1^cre^IL-4Rα^-/lox^) deficient mice in a time-kinetic manner following *Mtb* infection. At 4 weeks post-infection, mycobacterial lung burdens in mb1^cre^IL-4Rα^-/lox^ mice were similar when compared to littermate control animals (**Figure 1A**). However, at 18 weeks post-infection, mycobacterial burdens in both lungs and spleen were significantly reduced in mb1^cre^IL-4Rα^-/lox^ mice when compared to littermate controls (**Figure 1B**). Furthermore, we determined the lung inflammation by H&E and performed immunohistochemistry for iNOS expression. At 4- and 18-weeks post-infection, we found that pulmonary pathology, lesion area (**Figures 1C, D**) and iNOS expression (**Figures 1C, E**) were significantly decreased in mb1^cre^IL-4Rα^-/lox^ mice, indicating reduced lung tissue destruction during *Mtb* infection. These results show that B cell-specific IL-4Rα ablation decreased mycobacterial burden, lung inflammation and iNOS expression in mb1^cre^IL-4Rα^-/lox^ mice during chronic tuberculosis.

### Adoptive Transfer of IL-4Rα-Sufficient B Cells Abolished Decreased *Mtb* Burdens and Lung Pathology in mb1^cre^IL-4Rα^-/lox^ Mice

**Figure 2:**
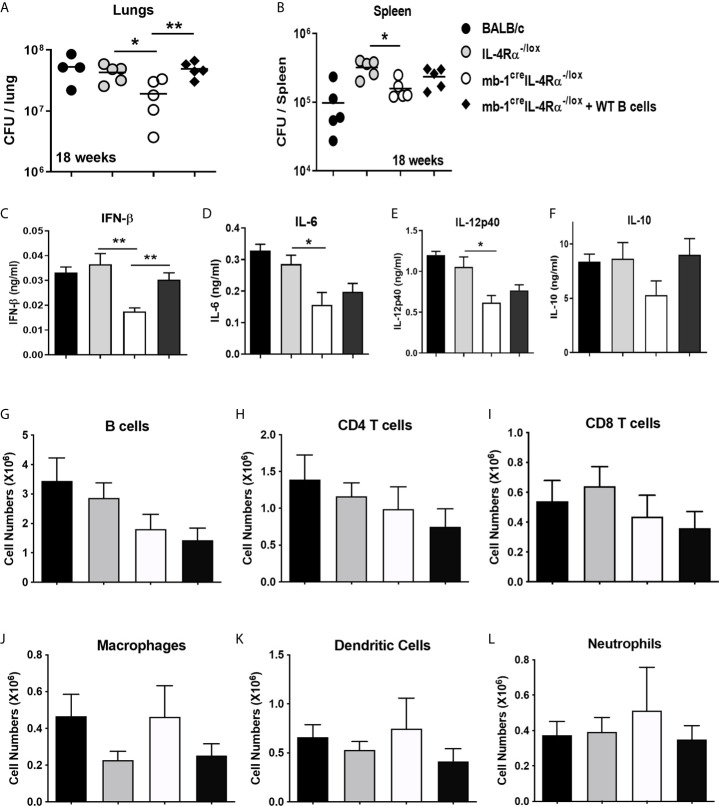
Adoptive transfer of WT B cells intratracheally restored the bacterial burdens in B cell-specific IL-4Rα deficient mice during *Mtb* infection. Wild-type (BALB/c), littermate controls (IL-4Rα^-/lox^), B cell-specific IL-4Rα deficient mice (mb1^cre^IL-4Rα^-/lox^) and adoptively transferred B cells in mb1^cre^IL-4Rα^-/lox^ mice (mb1^cre^IL-4Rα^-/lox^ + WT B cells) were infected intranasally with a 375CFU of H37Rv. **(A, B)** Bacterial burdens in the lungs and spleen after 18 weeks post-infection. **(C–F)** Lung homogenates were analysed for the cytokine responses such as for **(C)** IFN-β, **(D)** IL-6, **(E)** IL-12p40 and **(F)** IL-10 cytokine production by ELISA. Single cell suspension of lung cells was analysed for lymphoid **(G–I)** and myeloid **(J–L)** immune cell populations by flow cytometry. Cells were identified using the markers in parathesis; B cells (CD19^+^CD3^-^), CD4 T cells (CD3^+^CD4^+^), CD8 T cells (CD3^+^CD8^+^), dendritic cells (CD11c^+^CD11b^-^MHCII^+^), macrophages (CD11b^+^CD11c^-^MHCII^+^) and neutrophils (SiglecF^-^CD11c^-^Gr1^+^). Data are shown as mean ± SEM of n = 5 mice/group, representative of two independent experiments, analysed by unpaired, student’s t-test versus littermate control, *p < 0.05 and **p < 0.01.

**Figure 3:**
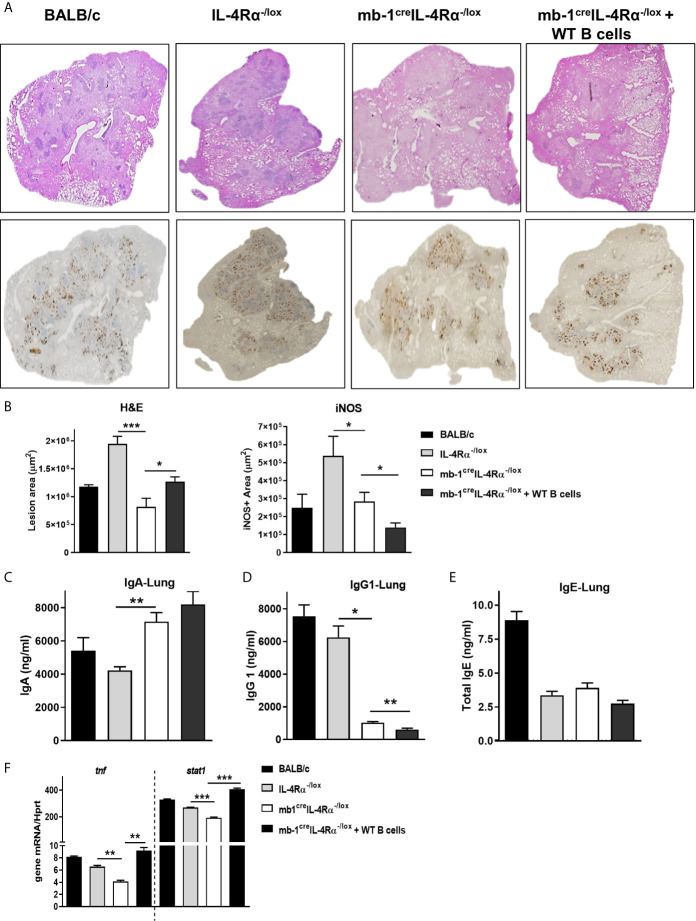
Adoptive transfer of WT B cells in the lungs restored the pulmonary pathology in B cell-specific IL-4Rα deficient mice during *Mtb* infection. Formalin-fixed lung samples were stained for the H&E and iNOS expression after 18 weeks post-infection. **(A)** Representative histology images from all the groups and **(B)** quantification of lesions area and iNOS positive staining in the lungs (Original magnification: 2X). Antibody responses in the lungs of mice. **(C)** IgA, **(D)** IgG1 and **(E)** total IgE production in the lungs after 18 weeks of infection. **(F)** *Tnf* and *Stat1* mRNA expression in flow-sorted B cells (CD3^-^CD19^+^B220^+^) after 18 weeks of *Mtb* infection. Data are shown as mean ± SEM of n = 5 mice/group, representative of two independent experiments, analysed by unpaired, student t-test versus littermate control, *p < 0.05, **p < 0.01 and ***p < 0.001.

We investigated whether the host-protective phenotype in mb1^cre^IL-4Rα^-/lox^ mice was indeed B cell-driven. To this end, we adoptively transferred one million wild-type B cells in mb1^cre^IL-4Rα^-/lox^ mice intratracheally followed by *Mtb* infection. At 18 weeks post-infection, we found the transfer of wild-type B cells restored the lung mycobacterial burdens similar to littermate control animals (**Figure 2A**). Though the spleen mycobacterial burden was partially restored but not statistically significant, this is likely due to intratracheal B cell transfer rendered minor effect on the distal organ spleen (**Figure 2B**). We then assessed the cytokine responses in the lung homogenates, which showed that IFN-β (**Figure 2C**), IL-6 (**Figure 2D**) and IL-12p40 (**Figure 2E**) was significantly reduced whereas IL-10 (**Figure 2F**) had no effect in mb1^cre^IL-4Rα^-/lox^ mice when compared to littermate control animals. Remarkably, the adoptive transfer of wild-type B cells restored IFN-β (**Figure 2C**) production, but not IL-6 (**Figure 2D**), IL-12p40 (**Figure 2E**) and IL-10 (**Figure 2F**) in the lungs of mb1^cre^IL-4Rα^-/lox^ mice. Given the significant differences in IFN-β, we assessed whether *Mtb* exposure of wild type B cells influences ifnb1 mRNA expression levels. We found no difference in ifnb1 mRNA expression in *Mtb*-exposed B cells when compared to naïve cells (**Supplementary Figure 1A**). Furthermore, we flow-sorted lung B cells from chronic *Mtb*-infected mb1^cre^IL-4Rα^-/lox^ mice, which showed no difference in ifnb1, il10 and il6 mRNA expression when compared to B cells from control animals (**Supplementary Figure 1B**). These findings indicated that B cells indirectly regulate IFN-β production. IFN-β can regulate anti-inflammatory responses by inducing IL-10 expression in the context of LPS stimulated and *Mtb* infected macrophages (32, 33). However, intracellular cytokine staining revealed IL-10-producing B cells were unaffected in chronic *Mtb*-infected mb1^cre^IL-4Rα^-/lox^ mice (**Supplementary Figure 1C**). We then assessed lymphoid and myeloid immune cell populations in the lungs by flow cytometry. We found no difference in B cells (**Figure 2G**), CD4 (**Figure 2H**), CD8 T cells (**Figure 2I**), macrophages (**Figure 2J**), dendritic cells (**Figure 2K**) and neutrophils (**Figure 2L**) in the lungs of mb1^cre^IL-4Rα^-/lox^ mice. Moreover, we further analysed the B cell subsets in the lungs of *Mtb*-infected mice. We found that except CD43^-^IgM^+^ B cells, deletion of IL-4Rα had no effect on B-1a, B-1b, B-2, B-10, plasma cells, plasmablast and IgD^+^ B cell populations when compared to control animals (**Supplementary Figures 1D, E**). We then assessed the impact of adoptively transferred wild-type B cells on lung pathology (H&E) and iNOS expression. Indeed, wild-type B cells restored lung pathology (**Figure 3A**) and lesion area (**Figure 3B**) similar to control animals but iNOS expression was unchanged (**Figures 3A, B**) in mb1^cre^IL-4Rα^-/lox^ mice. This indicates that B cells do contribute to the lung pathology independent of iNOS expression. Together, these results suggest that intact IL-4Rα on B cells contribute to mycobacterial burdens with lung pathology with no major impact on B cell subsets in chronic tuberculosis infection.

### IL-4Rα Deletion Modulates Antibody Production in the Lungs of mb1^cre^IL-4Rα^-/lox^ Mice

We then explored the influence of IL-4Rα deletion on antibody responses in the lungs during *Mtb* infection. At 18 weeks post-infection, we found increased protective IgA (**Figure 3C**) in the lung homogenates. The quantification of IgA positive areas in lung sections by immunohistochemistry further confirmed our findings of IgA in the lung homogenates (**Supplementary Figure 1G**). Permissive IgG1 (**Figure 3D**) production was decreased and total IgE (**Figure 3E**) remained unaffected in the lungs of mb1^cre^IL-4Rα^-/lox^ mice when compared to littermate control animals. IL-4 is the first identified stimuli that induce IgG1 production through isotype class switching by germ-line transcript induction (34). Sterile transcripts of IgG1 in B cells stimulated with LPS and IL-4 showed a trend of decreased production germline IgG1 transcripts in splenic B cells isolated from mb1^cre^IL-4Rα^-/lox^ mice (**Supplementary Figure 1F**). Moreover, adoptively transferred B cells had no major impact on the antibodies in the lungs when compared to mb1^cre^IL-4Rα^-/lox^ mice, except IgG1 levels which interestingly further decreased (**Figure 3D**). These results suggest that IL-4Rα signalling does modulate B cell antibody responses in the lungs. To better understand the B cell responses at the molecular level, we sorted B cells from the lungs of *Mtb*-infected mice to perform quantitative PCR after 18 weeks of infection. B cells showed reduced mRNA transcripts of *tnf* and *stat1* in mb1^cre^IL-4Rα^-/lox^ mice, which was increased similar to control animals (**Figure 3F**) following adoptive transfer of B cells. This points towards a reduced Be1 signature in mb1^cre^IL-4Rα^-/lox^ mice. Despite decreased levels of *stat1* mRNA expression in B cells, we found similar levels of IFN-γ in the lungs (data not shown), suggesting that T cells and NK cells may contribute to the production of IFN-γ. Together, these results suggest that the deletion of IL-4Rα on B cells modulates lung antibody responses and decrease *tnf* and *stat1* mRNA expression in mb1^cre^IL-4Rα^-/lox^ mice during chronic tuberculosis infection.

### Deletion of IL-4Rα on B Cells Reduced Association With *Mtb*

**Figure 4:**
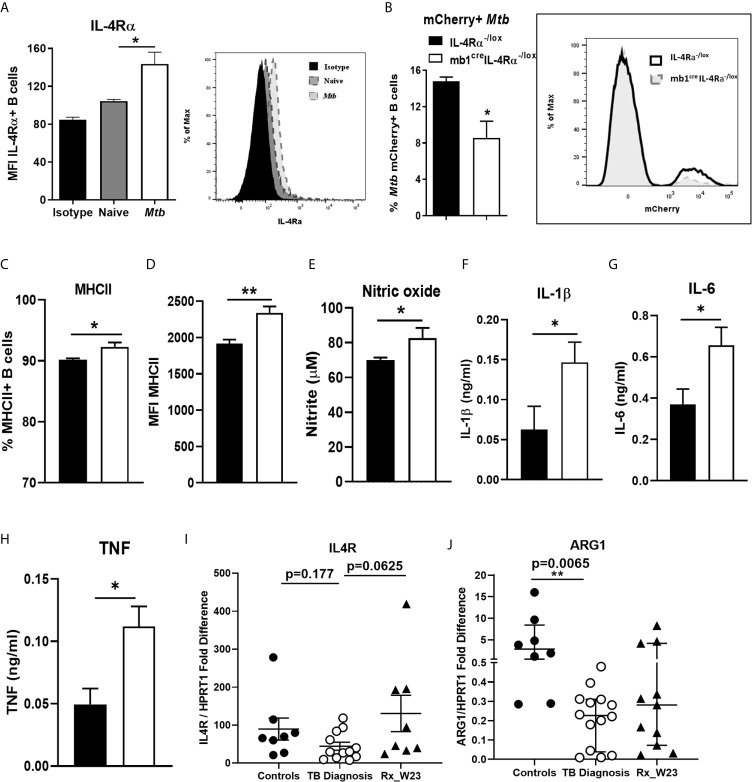
*Mtb* infection of B cells from mb1^cre^IL-4Rα^-/lox^ mice induces pro-inflammatory responses in macrophages. B cells were purified by magnetic bead sorting from the spleen of naïve wild-type mice. B cells were then infected with H37Rv *Mtb* (MOI=2) for 24 hours. **(A)** IL-4Rα surface expression measured in naïve and *Mtb* exposed B cells by flow cytometry. Magnetic bead sorted B cells from IL-4Rα^-/lox^ and mb1^cre^IL-4Rα^-/lox^ mice were infected with mCherry-expressing *Mtb* for 24 hours. **(B)** mCherry-expressing *Mtb* and **(C, D)** MHC II expression on B cells from IL-4Rα^-/lox^ and mb1^cre^IL-4Rα^-/lox^ mice were analysed by flow cytometry. **(E)** *Mtb*-infected macrophages were cultured with the supernatants from the *Mtb* infected B cells for 72 hours. Supernatants were then analysed for the production of **(E)** nitric oxide, **(F)** IL-1β **(G)** IL-6 and **(H)** TNF by ELISA. Data are shown as mean ± SEM of n = 3 mice/group and representative of three independent experiments, analysed by unpaired, student t-test versus littermate control, *p < 0.05, **p < 0.01. **(I)** Human IL4R mRNA and **(J)** Arginase 1 expression was determined in magnetic bead sorted B cells from healthy, TB patients at diagnosis and after 23 weeks of anti-TB therapy by qPCR with p values between the indicated groups (n=8-12 samples) analyzed by one-way ANOVA, **p < 0.01 versus controls.

We further investigated whether B cells increase IL-4Rα expression upon *Mtb* infection *ex vivo*. Magnetic bead-sorted wild-type B cells showed that *Mtb* infection significantly increased IL-4Rα expression when compared to naïve B cells after 24 hours (**Figure 4A**), which was further confirmed by qPCR (**Supplementary Figure 1H**). We then asked whether *Mtb* may associate differentially with B cells from IL-4Rα^-/lox^ and mb1^cre^IL-4Rα^-/lox^ mice. Indeed, flow cytometry revealed the frequency of mCherry-expressing *Mtb* positive cells was reduced in B cells from mb1^cre^IL-4Rα^-/lox^ mice after 24 hours (**Figure 4B**). Furthermore, we found increased MHCII positive B cells (**Figure 4C**) and expression (**Figure 4D**), indicating increased antigen presentation by the B cells from mb1^cre^IL-4Rα^-/lox^ mice when compared to controls. We then assessed the potential impact of *Mtb*-infected B cell supernatants on macrophages during infection. Following *Mtb* infection, macrophages were cultured with supernatants from *Mtb* exposed B cells from either mb1^cre^IL-4Rα^-/lox^ or IL-4Rα^-/lox^ mice. After three days, macrophages cultured with B cell supernatants from mb1^cre^IL-4Rα^-/lox^ mice showed a significant increase in nitric oxide (**Figure 4E**), IL-1β (**Figure 4F**), IL-6 (**Figure 4G**) and TNF (**Figure 4H**) production. Interestingly, IL-4Rα expression in B cells sorted from peripheral blood of TB patients at the diagnosis showed no difference in IL4R transcripts (**Figure 4I**) when compared to healthy controls. However, arginase 1 significantly decreased in blood B cells of TB patients (**Figure 4J**). Altogether, this indicate that IL-4Rα deletion on B cells does increase macrophage proinflammatory responses and their killing effector function.

## Discussion

Cytokine measurements in patients with TB suggested a role for IL-4Rα-driven T helper 2 immunity in the progression of the disease (35). IL-4 secretion in PBMC is elevated and involved in cavitary granuloma formation in patients with active TB disease (36, 37). Murine models using IL-4^-/-^, IL-4Rα^-/-^ and STAT6^-/-^ on genetically resistant C57BL/6 background proved to be dispensable in *Mtb* infection (21, 38, 39). Interestingly in C57BL/6 mice, transgenic expression of IL-13 uncovered that IL-13/IL-4Rα signaling contributes to TB-associated pathology (40). In contrast to overall C57BL/6, TB disease progression likely associated with Th2 immune response in BALB/c mice during chronic infection (41). Furthermore, immunotherapy using anti-IL-4 or anti-IL-13 or combined IL-4/IL-13 neutralizing antibodies (42) and high-dose *Mtb*-infected IL-4^-/-^ BALB/c mice resulted in decreased bacterial loads (39) and attenuated lung pathology (41). Together, data derived from BALB/c mice demonstrated that Th2 immune response contributes to disease progression, and therefore blocking IL-4 seems an attractive therapeutic approach (43). BALB/c mice appear to be a suitable model for investigating Th2 immunity in tuberculosis (44). Thus, we assessed whether depletion of IL-4Rα on B cells in mb1^cre^IL-4Rα^–/lox^ BALB/c mice in tuberculosis.

In addition to IL-4/IL-4Rα axis, B cells are critical in antibody production and they are efficient antigen-presenting cells. The success of antibodies in passive immunization suggested that certain antibodies are protective against TB (45). Mice lacking B cells showed relatively modest disease phenotypes during *Mtb* infection (19, 20). Moreover, B cell-deficient (IgH-6^-/-^) mice on a C57BL/6J background were dispensable in chronic tuberculosis (21). In acute tuberculosis, B cell-mediated humoral immunity is required to control inflammation and protective immunity (19, 22, 46). Surprisingly, B cell-deficient uMT^-/-^ mice infected with CDC1551 (22) strain of *Mtb* displayed similar burdens in the acute phase and reduced lung inflammation in chronic TB. In contrast, uMT^-/-^ mice showed enhanced mortality as a result of increased neutrophils and IL-10 production in the lungs in the Erdman strain of *Mtb* infection (19). Moreover, the non-human primate model of cynomolgus macaques showed that *Mtb*-containing granulomas are surrounded by proliferating B cells, secreting *Mtb*-specific (IgG) antibodies (15). However, B cell depletion using rituximab resulted in highly heterogeneous responses in local granuloma immune modulation, due to the antibody-dependent and -independent functions of B cells and altogether had no impact on the TB disease outcome (25). These studies highlighted the intricate role of B cells in TB disease stage and regulate the lung granulomatous response.

In wild-type mice, *Mtb* infection increased IL-4Rα expression on B cells and the absence of IL-4Rα on B cells decreased the frequency of *Mtb* infected B cells from mb1^cre^IL-4Rα^-/lox^ mice. There are limited studies on B cell internalization of bacteria; it has been shown that the human Raji B cell line can phagocytose complement opsonized *Mtb (* 47). Macropinocytosis can also be employed by immortalized B cells for the uptake of *Mtb (* 48). The effects of IL-4 signalling on phagocytosis are debatable as both increased and diminished phagocytic capacity were observed in macrophages (49–51). In the absence of IL-4Rα, we observed decreased internalization of *Mtb* by B cells; however, it warrants further studies on whether IL-4 alters phagocytic capacity and phagosome phenotype in B cells (52). Remarkably, genetic ablation of IL-4Rα on B cells (mb-1^cre^IL-4Rα^–/lox^) in mice showed reduced lung burdens and splenic dissemination in chronic tuberculosis infection. This was also accompanied by reduced lung pathology, lesion area and iNOS expression. In contrast, IL-4Rα deleted on macrophage/neutrophils, LysM^cre^IL-4Rα^-/lox^ mice had no differences in tissue bacterial burdens (29). Interestingly, the immune cell populations remained unaffected in mb1^cre^IL-4Rα^-/lox^ mice, which corroborated with our previous findings in LysM^cre^IL-4Rα^-/lox^ mice during tuberculosis (29). Characterization of lung B cell subsets also revealed no major differences between mb-1^cre^IL-4Rα^–/lox^ mice and littermate controls except IgM^+^IgD^-^CD43^-^ subset. These cells are either lung B1 cells expressing low levels of CD43 or distinct anergic, short-lived, B cell receptor unresponsive cells B2 cells (53, 54). In-depth phenotyping of this subset may explain whether IL-Rα signalling is important for the maintenance and the decreased numbers of these cells contribute to protection in mb-1^cre^IL-4Rα^–/lox^ mice. In contrast, B cell depletion (rituximab) in macaques, lead to increased T cell frequencies and cytokine responses unable to drive host protection during *Mtb* infection (25). These data suggest that IL-4Rα signalling on B cells modulate *Mtb* infection more at the site of infection in the chronic phase of tuberculosis.

Remarkably, the adoptive transfer of wild-type B cells in mb-1^cre^IL-4Rα^-/lox^ mice reversed lung bacterial burdens, lung pathology and lesion area similar to wild-type mice. The absence of B cells does not affect lung IFN-γ levels (19). This is likely compensated due to the release of IFN-γ from natural killer and T cells. In tuberculosis, increased levels of type I IFN is host detrimental (55). The decreased IFN-β production in mb-1^cre^IL-4Rα^-/lox^ mice in this study might be associated with reduced tissue pathology and lung bacterial burdens. These parameters were restored upon the adoptive transfer of wild-type B cells, suggesting intact IL-4Rα on B cells enhances or mediate disease pathology, independent of B cell-mediated IFN-β production. A recent study showed that *Mtb*-stimulated IL-4Rα-sufficient B cells drive alternative activation of macrophages through IFN-β production (56). However, the absence of IL4-Rα signalling on B cells does not seem to affect IFN-β levels on B cells in both *ex vivo Mtb* exposure or *in vivo* chronic *Mtb* infection. Therefore, it is plausible that IL-4Rα-deficient B cells will hinder alternative activation macrophage phenotype through other soluble factors. Indeed, deficiency of IL-4Rα on B cells increased macrophage ability to increase proinflammatory cytokines and nitric oxide production, indicating that IL-4Rα signalling on B cells modulate macrophage immune responses during *Mtb* infection. These animals also showed decreased lung IgG1 (host detrimental) and increased lung IgA (host protective) levels, which may partly contribute to protection against *Mtb* infection. Besides tuberculosis, mb-1^cre^IL-4Rα^-/lox^ mice during *N. brasiliensis* infection uncovered that IL-4Rα-responsive B cells-driven IL-13 and antigen processing contribute to T cell-mediated protective immunity (13). Furthermore, we demonstrated that IL-4Rα-responsive B cells are host detrimental against *Leishmania major* and host protective in *Schistosoma mansonii* infection. Mechanistically, we revealed a more general phenomenon that B cells regulate T cell polarization (10). Moreover, in *S. mansonii* infection, IL-4Rα-expressing B cells reduced egg-driven host detrimental tissue granulomatous inflammation *via* host protective IL-10 production in mice (57). In contrast, we found neither IL-10 nor evident regulation of T cell responses rather macrophage response modulation in mb1^cre^IL-4Rα^-/lox^ mouse model in TB, indicating the underlying mechanism is different and appears more local at the site of disease.

B cell proliferation increased in latent TB granuloma and decreased in an active TB granuloma. In humans, a study showed lower IL-4 expression in human B cells in circulation during TB infection (58). Consistently, we observed a lower trend in IL-4R and a significant decrease in arginase 1 mRNA expression in peripheral blood human B cells sorted from individuals diagnosed with TB pointing towards Be1 phenotype in this cohort. These findings further reinforce that blood may not be an appropriate compartment to explore the local tissue effect of B cells (56, 59). The importance of tissue site is further demonstrated where IFN-β production was unaffected in B cells isolated from peripheral blood but significantly upregulated in B cells from the pleural fluid (56). Therefore, B cells isolated from the lungs of TB patients will increase our current understanding of immune modulation at the tissue level. Overall, our study reveals the underappreciated role of IL-4Rα signalling on B cells during the chronic phase of tuberculosis infection in mice.

## Data Availability Statement

The raw data supporting the conclusions of this article will be made available by the authors, without undue reservation.

## Ethics Statement

Participant recruitment and follow-up were approved by the Human Research Ethics Committee of Stellenbosch University (N10/01/013). Written informed consent was obtained from all study participants. The patients/participants provided their written informed consent to participate in this study. The protocol was approved by the Animal Ethics Committee (AEC Permit Number: 015/040), Faculty of Health Sciences, University of Cape Town, Cape Town, South Africa.

## Author Contributions

SP, MO, IR, and AL: designing research studies, conducting experiments, human samples and analysis. SP, MO, MH, JC, RG, and RK: acquiring data and analyzing data. SP and MO: writing the manuscript. FB: resources and funding for the research. All authors contributed to the article and approved the submitted version.

## Funding

This work was supported by the ICGEB Arturo Falaschi, Claude Leon Foundation and CIDRI post-doctoral fellowship(s) to SP. ICGEB Arturo Falaschi post-doctoral and EDCTP post-doctoral fellowship(s) to MO. National Research Foundation (NRF), Oppenheimer Memorial Trust and Carnegie Corporation PhD Scholarships to MH. South African Medical Research Council (SAMRC) Unit on Immunology of Infectious Diseases, National Research Funding (NRF) South Africa and the South African Research Chair Initiative (SARChi) to FB. NRF South Africa Competitive Support for Unrated Researchers (CSUR) funding to AL. The research conducted using BSL3 equipment platform supported by core funding from the Wellcome Trust (203135/Z/16/Z).

## Conflict of Interest

The authors declare that the research was conducted in the absence of any commercial or financial relationships that could be construed as a potential conflict of interest.

## Supplementary Material

Supplementary Figure 1 Deletion of IL-4Rα on B cells had no impact on cytokine genes expression and B cell subsets. **(A)** Magnetic-bead sorted B cells from the spleen of naïve mice were exposed to *Mtb* (MOI=2) for 24 hours. *Ifnb* mRNA expression was determined by qPCR. Wild-type (BALB/c), littermate controls (IL-4Rα^-/lox^) and B cell-specific IL-4Rα deficient mice (mb-1^cre^IL-4Rα^-/lox^) were infected intranaslly with a dose of 150 CFU H37Rv. **(B)** Flow-sorted B cells (CD19^+^B220^+^CD3^-^) were analysed for *Ifnb1, il10* and *il-6* transcripts by qPCR. **(C)** The frequency of IL-10-producing B cells after 10 hours of PMA (20ng/ml)/ionomycin (1µg/ml) stimulation was determined by intracellular cytokine assay after 12 weeks post-infection. **(D)** B-1a (CD19^+^B220^+^CD43^+^CD5^high^IgM^high^), B1b (CD19^+^B220^+^CD43^+^CD5^low^IgM^high^), B2 (CD19^+^B220^+^CD43^-^IgM^+^IgD^+^) B10 (CD19^+^B220^+^CD43^+^CD5^+^CD1d^+^) and IgM (CD19^+^B220^+^CD43^-^IgM^+^IgD^-^). **(E)** Plasma (CD19^+^B220^+^ CD138^+^MHCII^low^CD44^high^), Plasmablast (CD19^+^B220^+^CD138^+^MHCII^high^CD44^high^) and IgD (CD19^+^B220^+^IgD^+^) B cells in the lungs of mice after 12 weeks of *Mtb* infection. **(F)** Germline transcript of *IgG1* measured by qPCR in splenic B cells stimulated with LPS (10ug/ml)/IL-4 (25ng/ml) for 48 hours. **(G)** Representative images and quantification of IgA-positive area of lung sections at 18 weeks post-infection (Original magnification: 40X). **(H)** B cells either left alone or infected with *Mtb* to determine IL-4Rα mRNA expression by qPCR. Data are shown as mean ± SEM of n = 6 mice/group, analysed by unpaired, student t-test or ordinary one-way ANOVA versus the indicated group, *p<0.05, **p<0.01 and ***p<0.001.

Supplementary Figure 2 Gating strategy for B cell subset identification in chronic *Mtb*-infected lungs of mice.

Supplementary Figure 3 Gating strategy for various immune cell populations in chronic *Mtb*-infected lungs of mice.

Supplementary Table 1 The list of antibody fluorophores and clones.

Supplementary Table 2 The list of primer sequences used for qPCR.
