# Supplementary figures and images for: IL-4-Responsive B Cells Are Detrimental During Chronic Tuberculosis Infection in Mice
Source: Front Immunol. 2021 Jun 15;12:611673. doi: 10.3389/fimmu.2021.611673 (PMC8243286; doi:10.3389/fimmu.2021.611673)

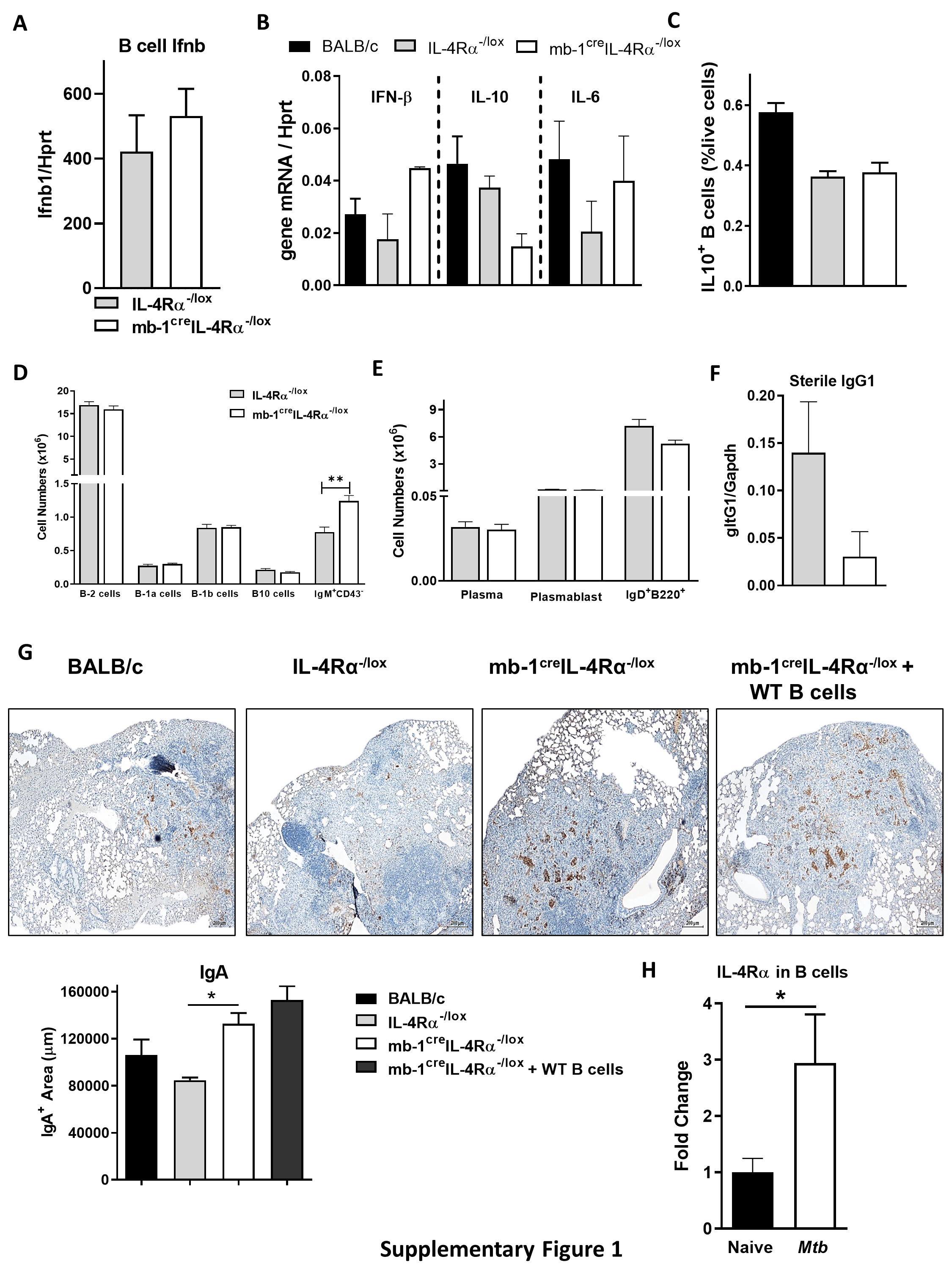

Supplement: Supplementary Figure 1 — Deletion of IL-4Rα on B cells had no impact on cytokine genes expression and B cell subsets. (A) Magnetic-bead sorted B cells from the spleen of naïve mice were exposed to Mtb (MOI=2) for 24 hours. Ifnb mRNA expression was determined by qPCR. Wild-type (BALB/c), littermate controls (IL-4Rα-/lox) and B cell-specific IL-4Rα deficient mice (mb-1creIL-4Rα-/lox) were infected intranaslly with a dose of 150 CFU H37Rv. (B) Flow-sorted B cells (CD19+B220+CD3-) were analysed for Ifnb1, il10 and il-6 transcripts by qPCR. (C) The frequency of IL-10-producing B cells after 10 hours of PMA (20ng/ml)/ionomycin (1µg/ml) stimulation was determined by intracellular cytokine assay after 12 weeks post-infection. (D) B-1a (CD19+B220+CD43+CD5highIgMhigh), B1b (CD19+B220+CD43+CD5lowIgMhigh), B2 (CD19+B220+CD43-IgM+IgD+) B10 (CD19+B220+CD43+CD5+CD1d+) and IgM (CD19+B220+CD43-IgM+IgD-). (E) Plasma (CD19+B220+ CD138+MHCIIlowCD44high), Plasmablast (CD19+B220+CD138+MHCIIhighCD44high) and IgD (CD19+B220+IgD+) B cells in the lungs of mice after 12 weeks of Mtb infection. (F) Germline transcript of IgG1 measured by qPCR in splenic B cells stimulated with LPS (10ug/ml)/IL-4 (25ng/ml) for 48 hours. (G) Representative images and quantification of IgA-positive area of lung sections at 18 weeks post-infection (Original magnification: 40X). (H) B cells either left alone or infected with Mtb to determine IL-4Rα mRNA expression by qPCR. Data are shown as mean ± SEM of n = 6 mice/group, analysed by unpaired, student t-test or ordinary one-way ANOVA versus the indicated group, *p<0.05, **p<0.01 and ***p<0.001. [file Image_1.tif]

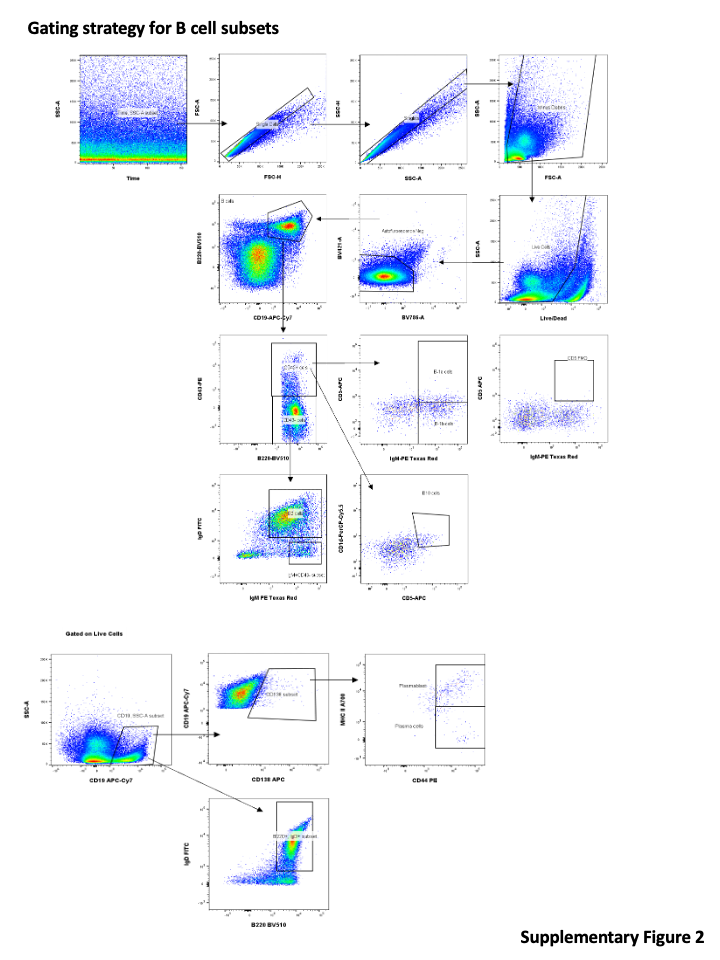

Supplement: Supplementary Figure 2 — Gating strategy for B cell subset identification in chronic Mtb-infected lungs of mice. [file Image_2.tiff]

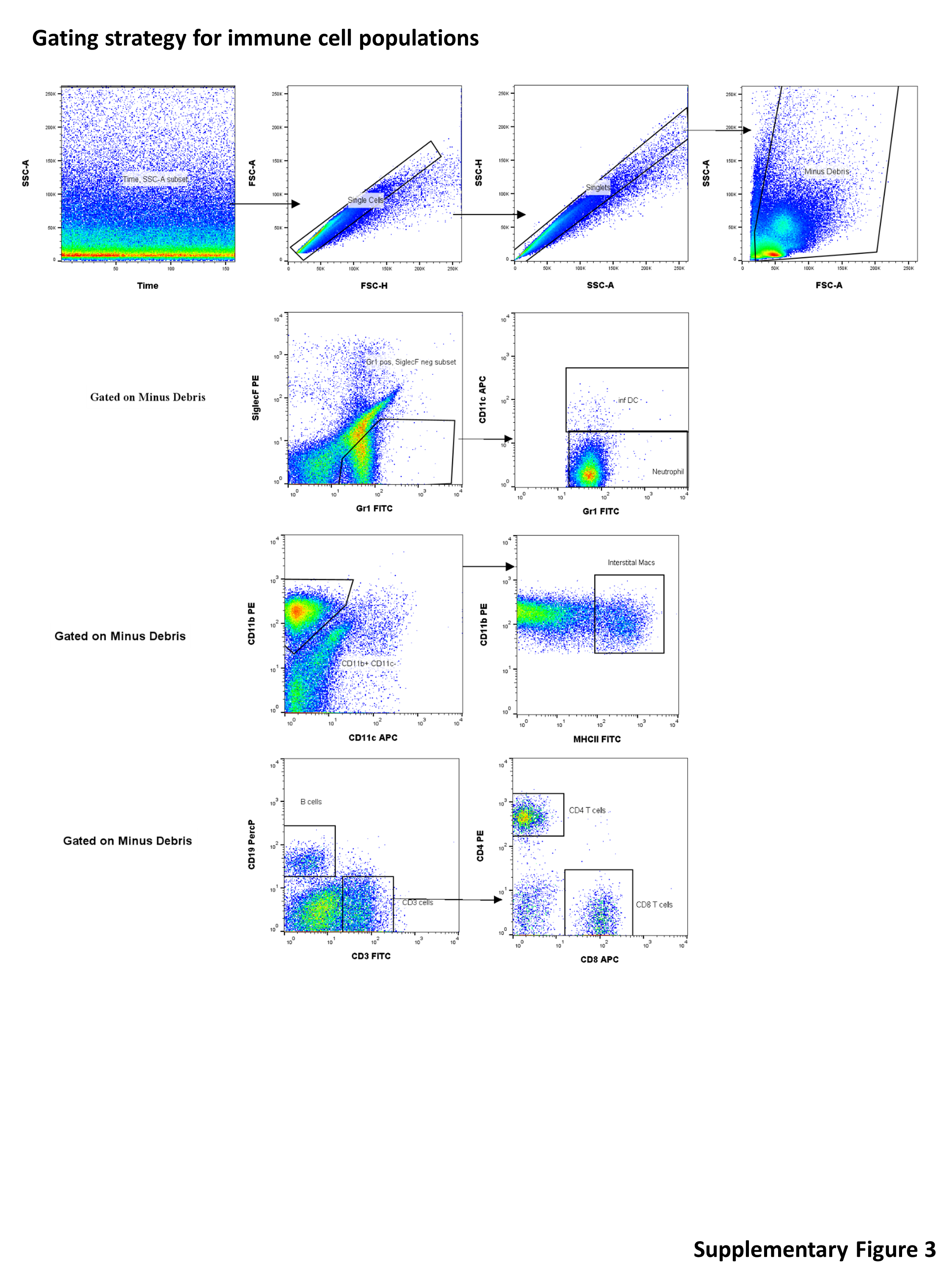

Supplement: Supplementary Figure 3 — Gating strategy for various immune cell populations in chronic Mtb-infected lungs of mice. [file Image_3.tif]
