# Supplementary material for: IL-4-Responsive B Cells Are Detrimental During Chronic Tuberculosis Infection in Mice
Source: Front Immunol. 2021 Jun 15;12:611673. doi: 10.3389/fimmu.2021.611673 (PMC8243286; doi:10.3389/fimmu.2021.611673)
Supplement: Supplementary Table 1 — The list of antibody fluorophores and clones. [file Table_1.pdf]

Supplementary Table 1: Antibody details

Target Protein	Conjugation	Clone	Company
B220	BV510	RA3-6B2	Biolegend
CD3	AF700	500A2	Biolegend
CD3	FITC	500A2	BD Biosciences
CD4	PE	RM4-5	Biolegend
CD8	APC	53-6.7	Biolegend
CD19	APC-eFluor780	1D3	Invitrogen
CD19	PerCP-Cy5.5	1D3	BD Biosciences
CD11c	APC	HL3	BD Biosciences
CD11b	PE	M1/70	BD Biosciences
CD44	PE	IM7	Biolegend
CD124	PE/Biotin	mIL4R-M1	BD Biosciences
CD138	APC	281-2	BD Biosciences
MHC II	FITC	2G9	BD Biosciences
MHCII	AF700	M5/114.15.2	Invitrogen
Siglec F	PE	E50-2440	BD Biosciences
Gr-1	FITC	RB6-8C5	Biolegend
IgM	Biotin	RMM-1	Biolegend
CD5	APC	53-7.3	BD Biosciences
CD43	PE	S7	BD Biosciences
IgD	FITC	11-26c.2a	BD Biosciences
CD1d	PerCP-Cy5.5	1B1	BD Biosciences
Streptavidin	PE-Dazzle594	n/a	Biolegend
IL10	PE	JES5-1663	Invitrogen
