# Supplementary material for: IL-4-Responsive B Cells Are Detrimental During Chronic Tuberculosis Infection in Mice
Source: Front Immunol. 2021 Jun 15;12:611673. doi: 10.3389/fimmu.2021.611673 (PMC8243286; doi:10.3389/fimmu.2021.611673)
Supplement: Supplementary Table 2 — The list of primer sequences used for qPCR. [file Table_2.pdf]

Supplementary Table 2: qPCR primers

Gene	Sequences
Tnf forward	5’-TCTCATCAGTTCTATGGCCC-3’
Tnf reverse	5’-GGGAGTAGACAAGGTACAAC-3’
Stat1 forward	5’-CCATGTCTCCAGAGGAGTTTGAT-3’
Stat1 reverse	5’-GTCGCCAGAGAGAAATTCGTG-3’
Ifnb forward	5’-CAGCTCCAAGAAAGGACGAAC-3’
Ifnb reverse	5’-GGCAGTGTAACTCTTCTGCAT-3’
Il10 forward	5’-AGCCGGGAAGACAATAACTG-3’
Il10 reverse	5’-CATTTCCGATAAGGCTTGG-3’
Il6 forward	5’-CGTGGAAATGAGAAAAGAGTTGTG-3’
Il6 reverse	5’-ATCTCTCTGAAGGACTCTGGCT-3’
GLTg1 forward	5’-CAGCCTGGTGTCAACTAG-3’
GLTg1 reverse	5’-CTGTACATATGCAAGGCT-3’
Hprt forward	5’-GTTGGATATGCCCTTGAC-3’
Hprt reverse	5’-AGGACTAGAACACCTGCT-3’
Gapdh forward	5’-TTCACCACCATGGAGAAGGC-3’
Gapdh reverse	5’-GGCATGGACTGTGGTCATGA-3’
Hs_IL-4Ra forward	5’-CACCTATGCAGTCAACATTTGGA-3’
Hs_IL-4Ra reverse	5’-GATGCGGAGGGAGGGTTCTA-3’
Hs_Arg forward	5’-TCTACAAAACAGGGCTACTCTCA-3’
Hs_Arg reverse	5’-GAGCAAGTCCGAAACAAGCC-3’
Hs_Hprt forward	5’-AGGCGAACCTCTCGGCTTT-3’
Hs_Hprt reverse	5’-AAGACGTTCAGTCCTGTCCAT-3’
